# Airfoil leading edge blowing to control bow shock waves

**DOI:** 10.1038/s41598-020-79048-w

**Published:** 2020-12-14

**Authors:** Francisco Lozano, Guillermo Paniagua

**Affiliations:** Zucrow Laboratories, Purdue University, 500 Allison Road, West Lafayette, IN 47907 USA

**Keywords:** Aerospace engineering, Mechanical engineering

## Abstract

This manuscript presents a detailed characterization of active control of bow shock waves via leading edge injection, including subsonic coolant ejection and the appearance of Coanda effects. The flow phenomena occurring at steady and pulsating flow injection regimes were analyzed using steady and unsteady two-dimensional Reynolds-Averaged Navier Stokes, leading to a precise evaluation of the thermal load and drag reductions. Steady supersonic injection yields the largest abatement in thermal load and aerodynamic drag, while subsonic or fluctuating ones can also provide significant improvements at reduced cooling mass flow rates. Furthermore, a Coanda effect, causing a non-symmetric flow topology, was observed and analyzed for reduced injection port size. This Coanda effect is due to the sudden expansion happening from the injection port to the main flow and it causes the flow topology at the leading edge to become non-symmetric despite the complete symmetry of the problem. This is the first time in the literature such a phenomenon is documented for a supersonic airfoil leading edge injection. Furthermore, it enables the design of novel flow control strategies for the leading edge shock topology and flow structures in supersonic flows.

## Introduction

Shock waves topologies appearing in the vicinity of aerodynamic bodies immersed in supersonic flows abate the steady and transient aero-thermal performance. The induced thermomechanical loads constraint severely the work of designers^1^, which is vital towards the conception of more efficient supersonic flying vehicles, and supersonic internal passages, such as supersonic turbomachinery for alternative power generation cycles^2,3^.

**Figure 1:**
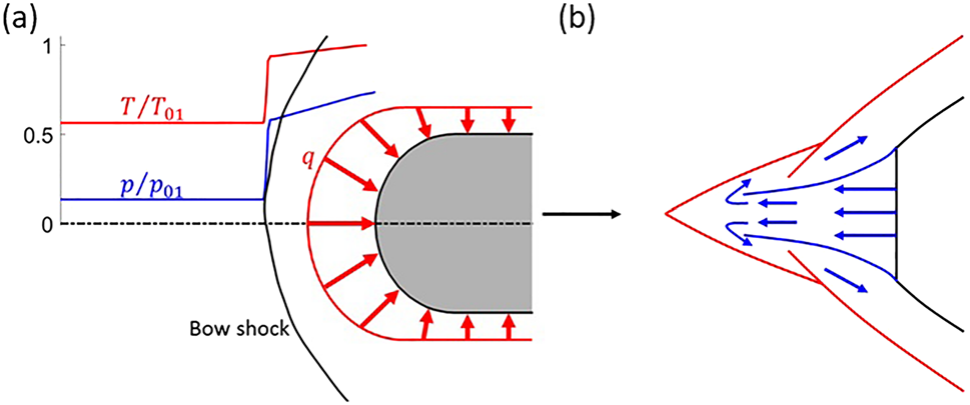
(**a**) Leading edge aerothermal challenge and (**b**) long penetration mode leading edge flow topology sketch, reproduced from Fomin et al.^11^.

The bow shock appearing at the leading edge region is particularly detrimental due to the high aerodynamic losses it creates, particularly due to its normal core region. Moreover, it is at the leading edge where the maximum thermal load is found for any geometry immersed in a supersonic stream. This aerothermal challenge is depicted in Fig. 1a. This graphic depicts the substantial increases in temperature and pressure suffered across the bow shock along the stagnation line, together with the leading edge heat flux distribution ($q$), exhibiting its maximum at the stagnation point.

A first approach to control it is the use of so-called aero-spikes or aero-disks^4,5^. These slender structures placed ahead of the leading edge modify the bow shock, turning its central normal region into two oblique shocks. This effect creates substantial reductions in wave drag. Nevertheless, it leads to severe aerodynamic and thermal loads on the aero-spike itself, limiting its practical implementation. A second approach relies on energy deposition, in which energy locally added upstream of the body creates a high temperature plasma bubble which interacts with the bow shock transforming its central region into two oblique shocks as well. The energy deposition methodology is proven to be successful in reducing drag^6,7^, yielding however a detrimental increase in the thermal load due to the rise it creates in the main flow total temperature.

Another strategy to control bow shock waves is the use of counterflowing jets injected at the leading edge region. There exists experimental and numerical evidence of the flow topology modifications this strategy creates, as well as the associated drag and heat flux reductions^8–10^. As the counterflowing jet merges with the mainstream, it modifies the leading edge shock pattern, which leads to a reduction in the pressure field over the body as well as pressure drag. Furthermore, the modulation of the boundary layer over the aerodynamic body created by the leading edge injection promotes a reduction in viscous drag. Fomin et al.^11^ studied the leading edge flow topology under different steady injection regimes through numerical inviscid simulations. at low injection pressures mainly the position of the bow shock is modified, pushing it upstream, with minor shape alteration, short penetration mode. There exists a pressure threshold beyond which the flow topology is dramatically changed, and the central part of the bow shock is replaced by two oblique shocks, like an aero-spike, long penetration mode. This flow topology is schematically depicted in Fig. 1b. Finley^12^ provided a detailed experimental characterization of the leading edge flow topology and wall pressure distributions for different flow injection intensities and leading edge geometries for the short penetration mode. Daso et al.^13^ also found the appearance of the short and long penetration modes using a combination of Reynolds-Averaged Navier Stokes (RANS) simulations and experimental studies. Interestingly, at extreme blowing rates, the bow shock disappears at the jet core^13^.

The flow control literature is prolific on test cases evaluating the airfoil trailing edge^14,15^or the leading edge^11^. However, the present manuscript explores for the first time in the literature the analysis of subsonic leading-edge ejection, using a steady and a pulsating stream. These new regimes require less energy than previous publications focused on supersonic ejection, abating some of the aerothermal penalties related to the leading edge bow shock. Furthermore, by exploring several ejection slot width relative to the leading edge diameter, we could identify a Coanda flow topology. Coanda flow, previously documented at a symmetric sudden expansion^16^ indicated the significance of unsteady 3D effects. To the authors’ knowledge, this work documents this phenomenon for the first time in supersonic leading edge bodies. The manuscript details the sudden flow ejection at different blowing ratios, providing relevant data for designers concerned with the cooling of supersonic aerodynamic bodies.

The approach employed for the present study is fully numerical, using steady and unsteady RANS. Different injection boundary conditions and geometries were assessed, evaluating their effect on the flow phenomena. The relation between modifications induced in these flow phenomena and the thermal load, drag and aerodynamic losses was evaluated in order to provide further tools to design more effective flow control and cooling strategies for supersonic aerodynamic bodies.

## Numerical methodology

### Computational domain

**Figure 2:**
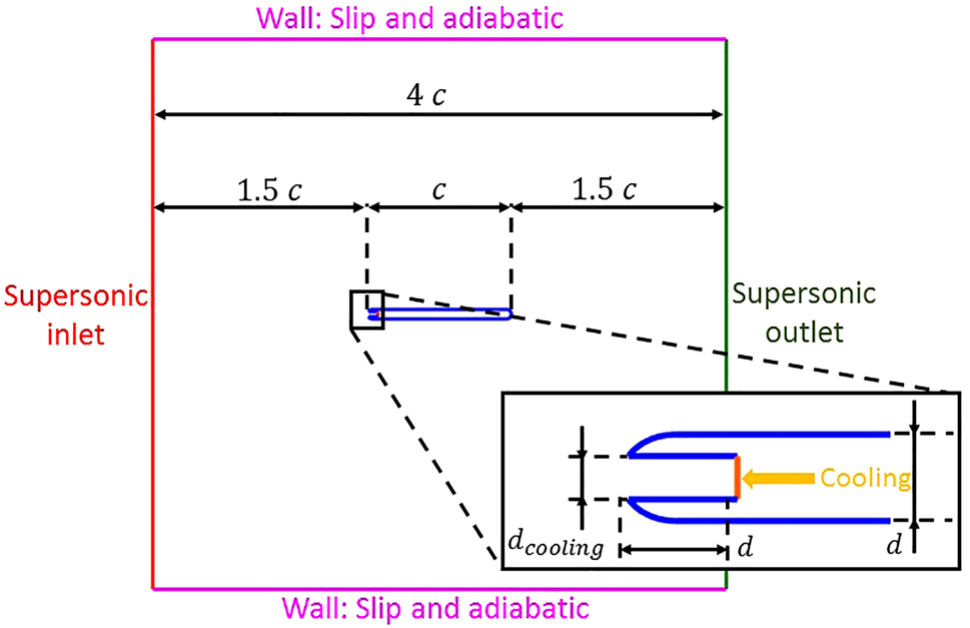
Computational domain and type of boundary conditions.

The computational domain is depicted in Fig. 2. It is a two-dimensional geometry consisting of a slender airfoil with a constant thickness $d = 4\,{\text{mm}}$ and semi-circular leading and trailing edge. The chord of the airfoil is $c = 60\,{\text{mm}} = 15 \times d$. The total axial length of the computational domain, illustrated in Fig. 2, is four times this axial chord, with the domain extended 1.5 times the chord upstream of the leading edge and 1.5 times downstream of the trailing edge. In the direction normal to the flow, the domain has a width $L_{y} = 230\,{\text{mm}} = 57.5 \times d$, with the airfoil being located at the center. Two different widths for the injection port were studied: $d_{cooling} = d/2$ and $d/10$.

### Boundary conditions

In Fig. 2, the flow moves from the left supersonic inlet to the right supersonic outlet. Total pressure and temperature as well as static pressure are imposed at the inlet, resulting in an inlet Mach number of $M_{1} = 1.98$. This main flow boundary conditions are given in Table 1. The outlet boundary condition, located on the right side of the computational domain, depicted in Fig. 2 is a supersonic outlet. Hence, the conditions at that section are not imposed but computed within the computational domain. The cooling injection is depicted in the zoomed leading edge area in Fig. 2. The coolant flow is injected opposing the mainstream in a cavity which is recessed one leading edge diameter. The boundary condition for the cooling injection is an inlet in which total pressure and temperature are imposed, together with static pressure in the supersonic injection cases. Regarding the unsteady simulations, at the coolant inlet boundary condition located at the leading edge, coolant total pressure varied in time while all the other boundary conditions remained the same as in the steady simulations. The unsteady blowing boundary conditions were prescribed through a coolant total pressure sinusoidal fluctuation, $p_{0\,cooling}$, with a constant total temperature. The mathematical expression describing the temporal evolution of $p_{0\,cooling} \,\left( t \right)$ is given in Eq. (1). Where $p_{0\,cooling\,mean}$ is the mean value of the cooling total pressure throughout the period. $A$ is the dimensionless peak-to-peak amplitude of the fluctuation as defined in Eq. (2), $p_{0\,cooling\,max}$ and $p_{0\,cooling\,min}$ correspond to the maximum and minimum values of cooling total pressure during a period.

**Table 1:** Main flow boundary conditions.

$p_{01} \,({\text{Pa}})$	757,749
$T_{01} \,({\text{K}})$	2743
$p_{1} \,({\text{Pa}})$	100,560
$T_{wall} \,({\text{K}})$	1000
$\alpha_{1} \,(^\circ )$	0

Table 2 presents the different cooling injection steady total pressures and temperatures investigated for cooling injection port sizes of $d/2$ and $d/10$.

1 $$ p_{0\,cooling} \,\left( t \right) = p_{0\,cooling\,mean} \left[ {1 + \frac{A}{2}\sin \left( {2\pi ft} \right)} \right] $$

**Table 2:**
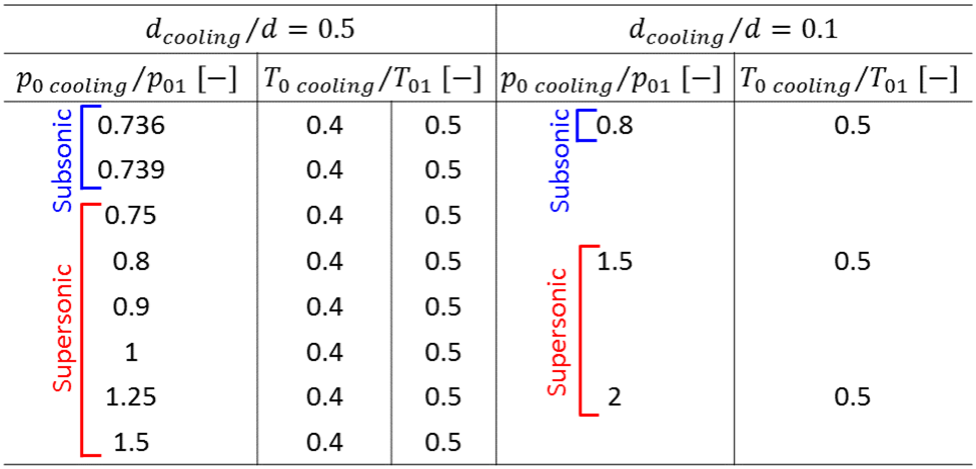
Cooling injection boundary conditions and geometry.

where

2 $$ A = \frac{{p_{0\,cooling\,max} - p_{0\,cooling\,min} }}{{p_{0\,cooling\,mean} }} $$

The maximum and minimum values for the cooling total pressure are such that $p_{0\,cooling\,max} /p_{01} = 0.75$ and $p_{0\,cooling\,min} /p_{01} = 0.736$. This gives a peak-to-peak amplitude $A = 1.9{\text{ \% }}$. The frequencies $\left( f \right)$ studied for the cooling total pressure fluctuations are 10, 100 and $1000\,{\text{Hz}}$. These have been studied for the two different cooling total temperatures ratios $0.4$ and $0.5$.

### Numerical approach

The numerical approach for the present study is based on two-dimensional steady and unsteady Reynolds-Averaged Navier Stokes (RANS and URANS) simulations for the cases with steady and unsteady actuation. The spatial discretization was performed using the commercial package Ansys ICEM. The solver used is Ansys Fluent, in its density-based mode in order to account for compressibility. Second-order upwind formulations were employed to solve the flow and turbulence transport equations. The transient solution for the unsteady simulations was solved with second-order implicit formulation.

The working fluid for both the main and coolant flow is air, modeled as an ideal gas, assuming constant specific heat capacity and thermal conductivity. Viscosity is modeled using Sutherland’s law. The turbulence closure was achieved using the k-$\omega$ SST model. All the simulations were performed considering the flow to be fully turbulent from the leading edge. This assumption is consistent with the recommendations from the turbine cooling community. According to Mayle^17^ and Dunn^18^, the injection of cooling at the leading edge triggers transition right at the leading edge, and therefore transition effects should be negligible. Note that in the present analysis, the inlet Reynolds number per unit length is ${\text{Re}}_{1,l} = 6.62 \times 10^{6} \,{\text{m}}^{ - 1}$. Two different grids are used throughout this study, for the cases with and without actuation. In both cases, the mesh is structured, and it is based on an O-grid that surrounds the airfoil. At the beginning of the study, a grid sensitivity analysis was performed for both grids, while monitoring: local distributions (pressure, heat flux and shear stress) at various positions along the airfoil; and integral quantities along the suctions and pressure side (heat flux integral along the airfoil walls and total drag). For all grids considered, a $y^{ + } < 1$ was always ensured at the airfoil walls guaranteeing a proper resolution of the boundary layer.

**Figure 3:**
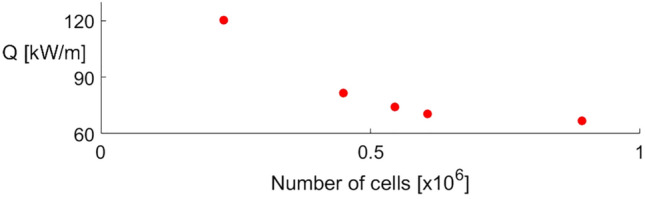
Thermal load evolution with respect to the number of cells for the different grids studied for the case with cooling injection.

A grid sensitivity study was performed for both cooled and uncooled cases. The grid was carefully selected to ensure the flow structures and wall variables are properly captured and grid-independent, despite the diffusive second-order upwind scheme, as observed in the experimental validation test case. In the case of leading-edge injection, five different meshes were considered. They contained 227,298, 449,406, 545,204, 605,459 and 891,976 cells. Figure 3 represents the evolution of the thermal load, i.e. the integral of the heat flux over the complete airfoil, with respect to the number of cells used in the spatial discretization. For the four grids studied other than the finest one, the relative error, $e_{a}$, as defined in Eq. (3), based on the work by Celik et al.^19^ has been evaluated. In that expression, $QoI$ represents a given quantity of interest. The relative error values for the thermal load are given in Table 3 for the thermal load. For the grid of 605,204 cells, $e_{a}^{Q}$ is reduced to a value of $5 \%$, while for drag this relative error is below $1 \%$ for all the grids. Furthermore, the differences between the local heat flux and wall shear stress between these two grids were also minor. This led to the choice of the grid with 605,204 cells as the discretization used for the rest of the study in the cases with leading edge injection. The same approach was followed for the mesh sensitivity study in the case of no actuation. The final chosen one has 267,316 cells.

3 $$ e_{a} = \left| {\frac{{QoI_{fine} - QoI}}{{QoI_{fine} }}} \right| $$

**Table 3:** Relative discretization errors from grid sensitivity study.

$N\,( - )$	227,298	449,406	545,204	605,459
$e_{a}^{Q} \,(\% )$	80	22	11	5

Note that all steady simulations were run for over 80,000 iterations to ensure convergence, ensuring that variations in relevant mass flows and quantities of interest such as the thermal load were below 0.05%. To ensure the Coanda flow is not a numerical artifact, cases yielding symmetric and asymmetric flow topologies were initialized from other symmetric and asymmetric solutions. In all cases, the symmetric or Coanda flow topology was always achieved independent of the simulation's initialization; the solution was only a function of the applied cooling boundary conditions.

For the unsteady cases, a time convergence study was performed as well. i.e. various time steps were studied to ensure that a finer time discretization would not change the physical phenomena captured by the simulations. This time convergence was assessed based on the temporal evolution of the airfoil thermal load, total pressure loss across the domain, cooling injection mass flow and Mach number. Periodic convergence of the simulations, i.e. how many periods were required to ensure periodic conditions, was assessed based on the work by Clark and Grover^20^ by evaluating the different harmonic amplitudes.

### Numerical approach validation

**Figure 4:**
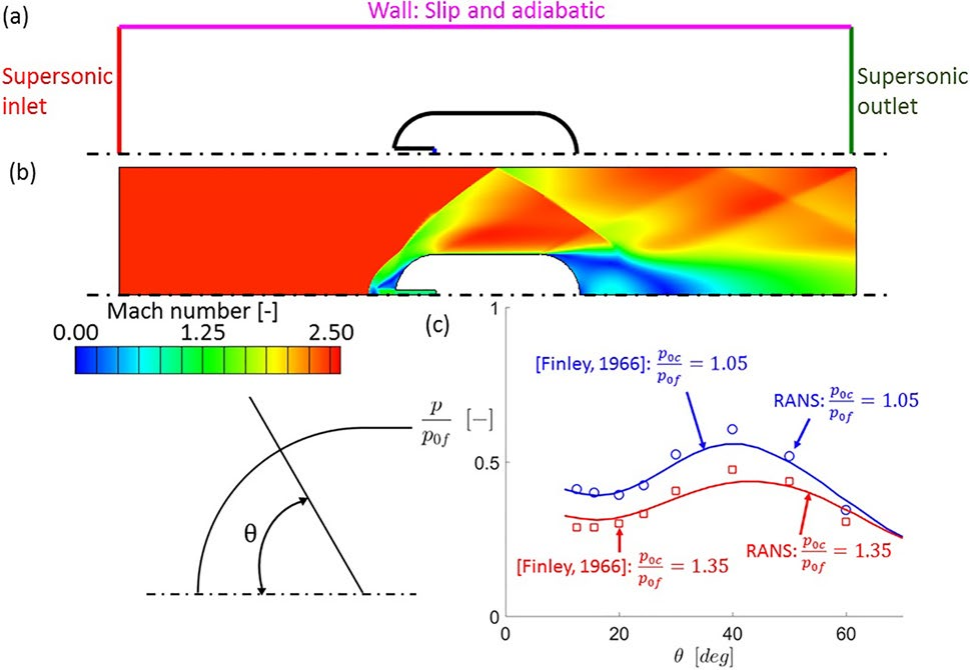
Validation case (**a**) computational domain and (b) Mach number contour for $p_{0c} /p_{0f} = 1.05$ and (**c**) leading edge experimental and numerical static pressure distributions.

The experimental work by Finley^12^ was employed to assess the validity of the 2D RANS methodology adopted in this study. Finley studied the flow phenomena occurring when a counterflowing jet is injected at the leading edge of a body facing a supersonic free stream. As a validation for the present study, one of his test articles, an axisymmetric body with a leading edge diameter of $50.8\,{\text{mm}} = 2 \,{\text{in}}$ and an injection port diameter of $7\,{\text{mm}}$, has been simulated. Figure 4a shows a schematic view of the computational domain for this validation case. An O-grid centered on the aerodynamic body like the one used for the rest of the study was employed. At the supersonic inlet on the left of the domain, the boundary conditions given in Table 4, which give a free stream Mach number of $2.5$, were imposed. The wall around the axisymmetric object were considered isothermal with a constant temperature of $300\,{\text{K}}$. Furthermore, a supersonic inlet with a Mach number of $1$ was imposed at the leading edge, with two different cooling total pressures such that $p_{0c} /p_{0f} = 1.05, 1.35$, where $p_{0c}$ is the cooling total pressure and $p_{0f}$ is the free stream Pitot pressure.

**Table 4:** Validation case main flow boundary conditions.

$p_{01} \,({\text{Pa}})$	275,790
$T_{01} \,({\text{K}})$	294
$p_{1} \,({\text{Pa}})$	16,141
$\alpha_{1} \,(^\circ )$	0

Figure 4b depicts the Mach number contour obtained for $p_{0c} /p_{0f} = 1.05$. The shock pattern like the one appearing for this injection will be analyzed in detail in the following section. Figure 4c depicts the static pressure distribution along the leading edge of the airfoil. On the horizontal axis, $\theta$ represents the angle with respect to the symmetry axis, while on the vertical axis the static pressure $p$ is normalized with the Pitot pressure. Solid lines correspond to the numerical data, while the discrete points correspond to the experimental results reported by Finley^12^. Near the symmetry axis, approximately for $\theta$ between $10^\circ$ and $20^\circ$, there is a region of low pressure in both cases, this correspond to a recirculation formed on the side of the injection port. As the angle $\theta$ increases, pressure increases towards a maximum located at the reattachment point^12^, decreasing after that as the flow expands downstream of the recirculation area. Numerical and experimental data show good agreement, with the maximum differences occurring at the reattachment point. These may be explained by the difficulty of RANS models to capture the reattachment of recirculation regions as shown by Saavedra and Paniagua^21^.

### Two- and three-dimensional flow topologies comparison

**Figure 5:**
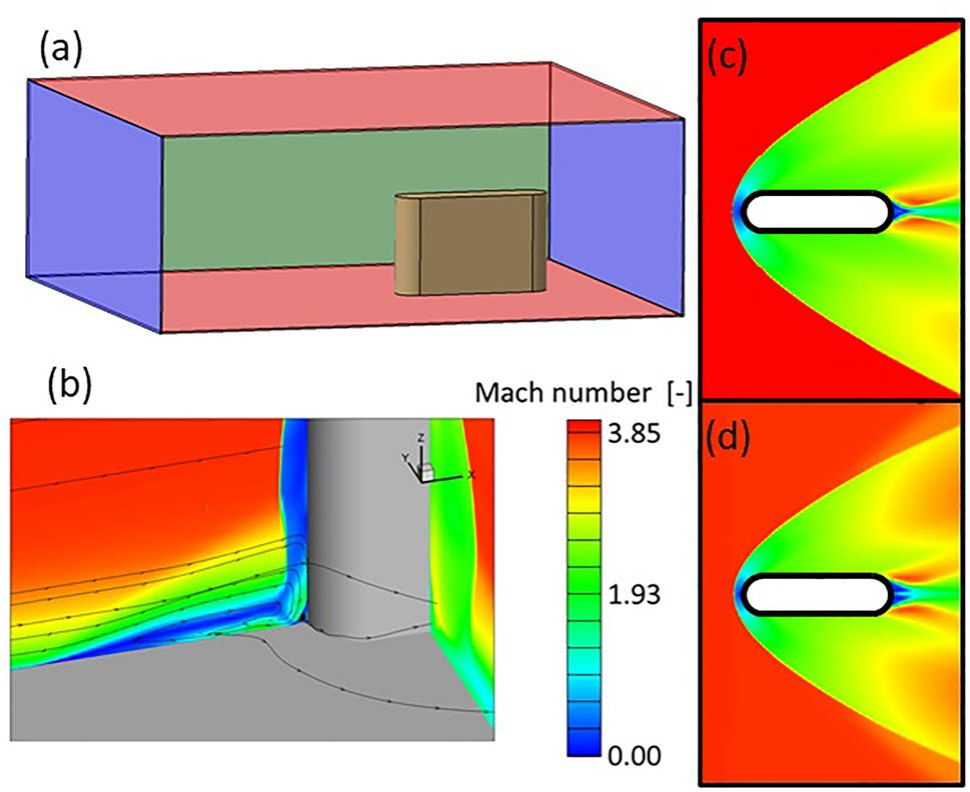
Computational domain (**a**), Mach contour slices and streamlines at 3D region at the leading edge (**b**), Mach contour corresponding to a 2D simulations (**c**), Mach contour cut on 2D region from 3D simulation (**d**) for a supersonic airfoil.

This manuscript presents a detailed analysis of the effects of flow control, considering a two-dimensional flow topology based on two-dimensional simulations. This section describes the spanwise flow field when the 2D cylindrical airfoil is mounted in a 3D configuration, attached to a bottom endwall. Figure 5a shows the computational domain used in the 3D RANS simulation. The blue surfaces designate the inlet and outlet sections, and the red surfaces correspond to the viscous bottom and top walls. The inlet boundary condition defines a supersonic flow at Mach 4, with a flat profile of total pressure and total temperature. The outlet boundary condition is a supersonic outlet. Figure 5b displays iso-Mach number contours, as well as streamlines. At the junction between the airfoil and the hub endwall, we observe a complicated 3D shock and the formation of a horseshoe vortex. However, moving away from that junction along the airfoil's span, a 2D topology is retrieved around the leading edge, dominated by the bow shock, consistent with studies available in the literature^22^. Figure 5c represents the Mach contour obtained from a 2D simulation with the same airfoil geometry and free stream conditions. Figure 5d depicts the iso-Mach levels extracted from the 3D simulation at a span of 80% of the total height of the domain. Both Figures (c) and (d) evidence identical flow topologies at the leading edge, with only minor differences on the trailing edge and outlet vicinity. In the present paper, the aim is to characterize the effect of flow control via leading-edge injection precisely in those regions where a 2D flow topology appears.

## Steady blowing effect

### Flow topology modifications

**Figure 6:**
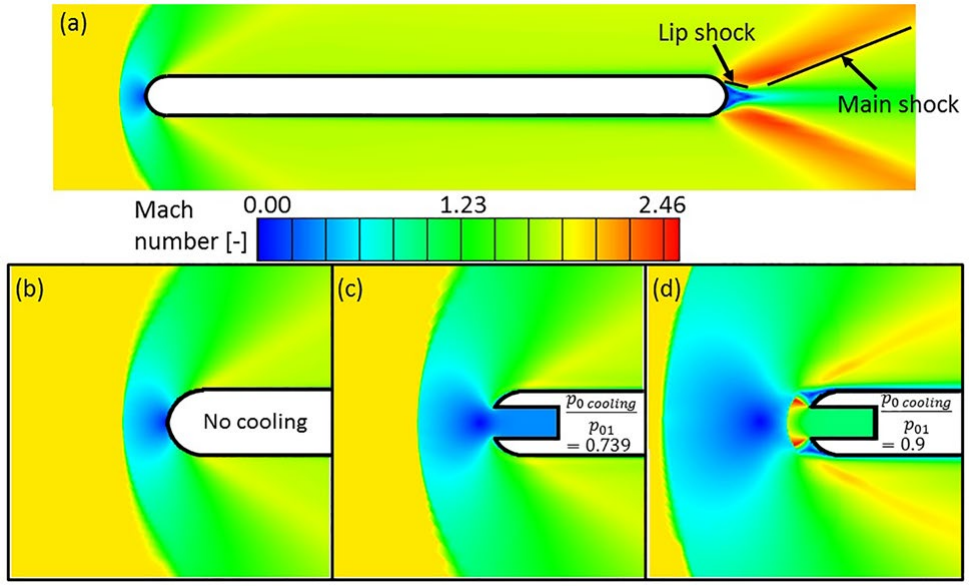
Mach number contours without cooling injection over the complete airfoil (**a**) and at the leading edge region (**b**) and with subsonic (**c**) and supersonic (**d**) injection at the leading edge region.

Figure 6a shows iso-Mach number levels around the airfoil for a case without coolant injection. The bow shock appearing upstream of the leading edge leads to a strong reduction in the Mach number. Further downstream, the flow reaccelerates due to the Prandtl–Meyer expansion fan appearing at the detachment point, followed by lip shocks and the main shock. When cooling is injected at the leading edge at injection total pressures $p_{0\,cooling} /p_{01} < 0.75$ the coolant is injected in a subsonic regime, for higher coolant stagnant pressures, a supersonic injection occurs. Two sample Mach contours of the leading edge region at these regimes are depicted in Fig. 6 at a cooling total temperature $T_{0\,cooling} /T_{01} = 0.5$.

For subsonic injection (see Fig. 6c), the Mach number in the injection vicinity remains below $1$, the cooling jet pushes the bow shock and the stagnation point upstream.

**Figure 7:**
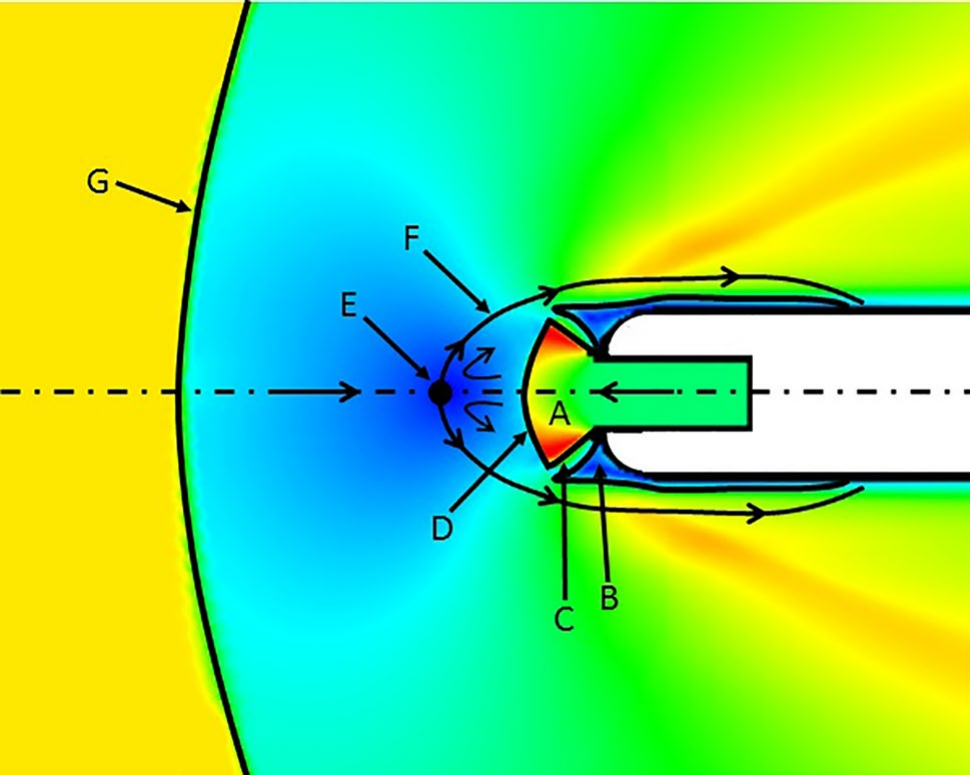
Leading edge region flow topology under supersonic injections conditions.

Figure 7 depicts a detailed sketch of the flow topology near the leading edge in supersonic injection conditions. The cooling flow achieves sonic conditions within the injection cavity. As soon as it departs from the cavity, it expands to supersonic conditions through a nozzle-shaped structure, region A. Region B corresponds to two recirculation regions appearing on opposite sides of the expansion region A. These two regions, A and B, are separated by shear layers (marked as C). The expansion region A is bounded upstream of the leading edge by a terminal shock (D) which defines the boundary between the supersonic region A and the subsonic region appearing upstream. Region G refers to the flow upstream of the bow shock. The saddle point (E) is found upstream of the terminal shock, where the cooling and main flow streams collide. Upstream of E the flow has the direction of the mainstream while downstream it has the direction of the opposing jet injected at the leading edge. As these two streams strike at the saddle point, they divert towards the upper and bottom sides of the airfoil forming an interface (F). The interface F divides the main high temperature stream from the opposing cooling stream, hence protecting the leading edge.

### Effect on aerodynamic performance

**Figure 8:**
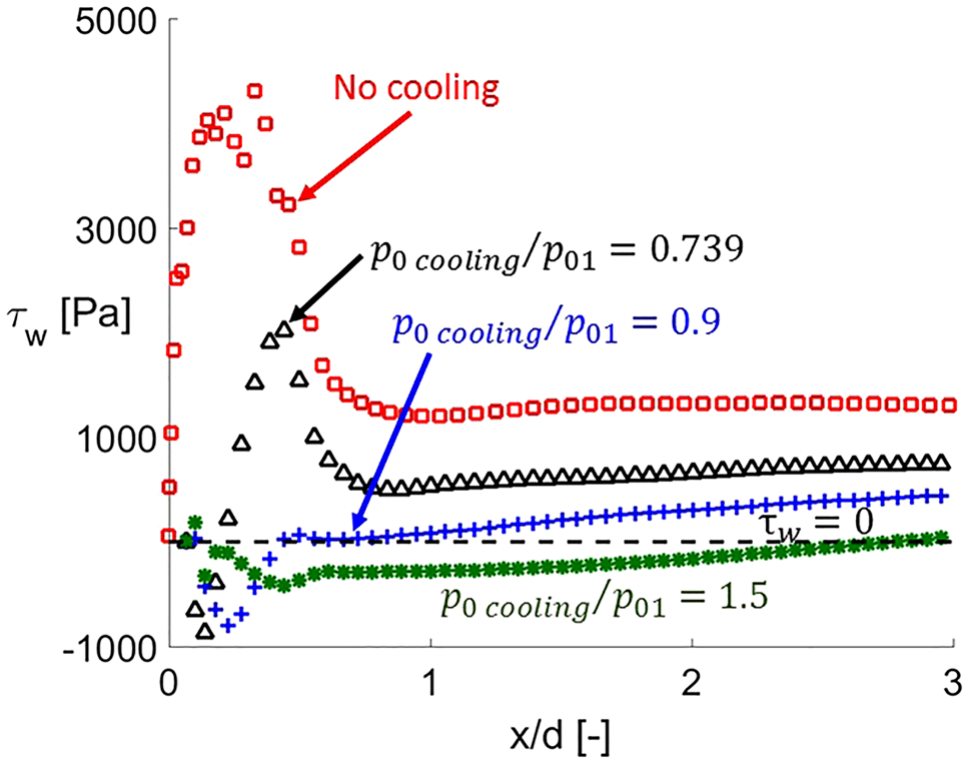
Wall shear stress distributions at the leading edge region under different cooling injection total pressures.

Figure 8 represents the shear stress signature for the different cases under study: no actuation, actuation cases at $T_{0\,cooling} /T_{01} = 0.5$ and $p_{0\,cooling} /p_{01}$ values from 0.739 to 1.5. In case of no injection, the wall shear stress distribution has a peak near the leading edge, where the flow accelerates after departing the stagnation point, before dropping towards a plateau.

However, for flow injection cases, the wall shear stress reflects negative values near the leading edge, identifying recirculated flow presence (B in Fig. 7). Once reattached the shear stress suddenly rises, mimicking the behavior of non-cooled case. As the cooling injection total pressure is increased, the extension of the separation region rises and the magnitude of the maximum wall shear stress appearing after decreases. In fact, for the case at $p_{0\,cooling} /p_{01} = 0.9$, the positive wall shear stress peak is suppressed. On both sides of the coolant ejection, the extension of the recirculation regions increases with the coolant injection total pressure. This more considerable extension of the separation region leads towards smoother density gradients, decreasing the wall shear after the reattachment point.

**Figure 9:**
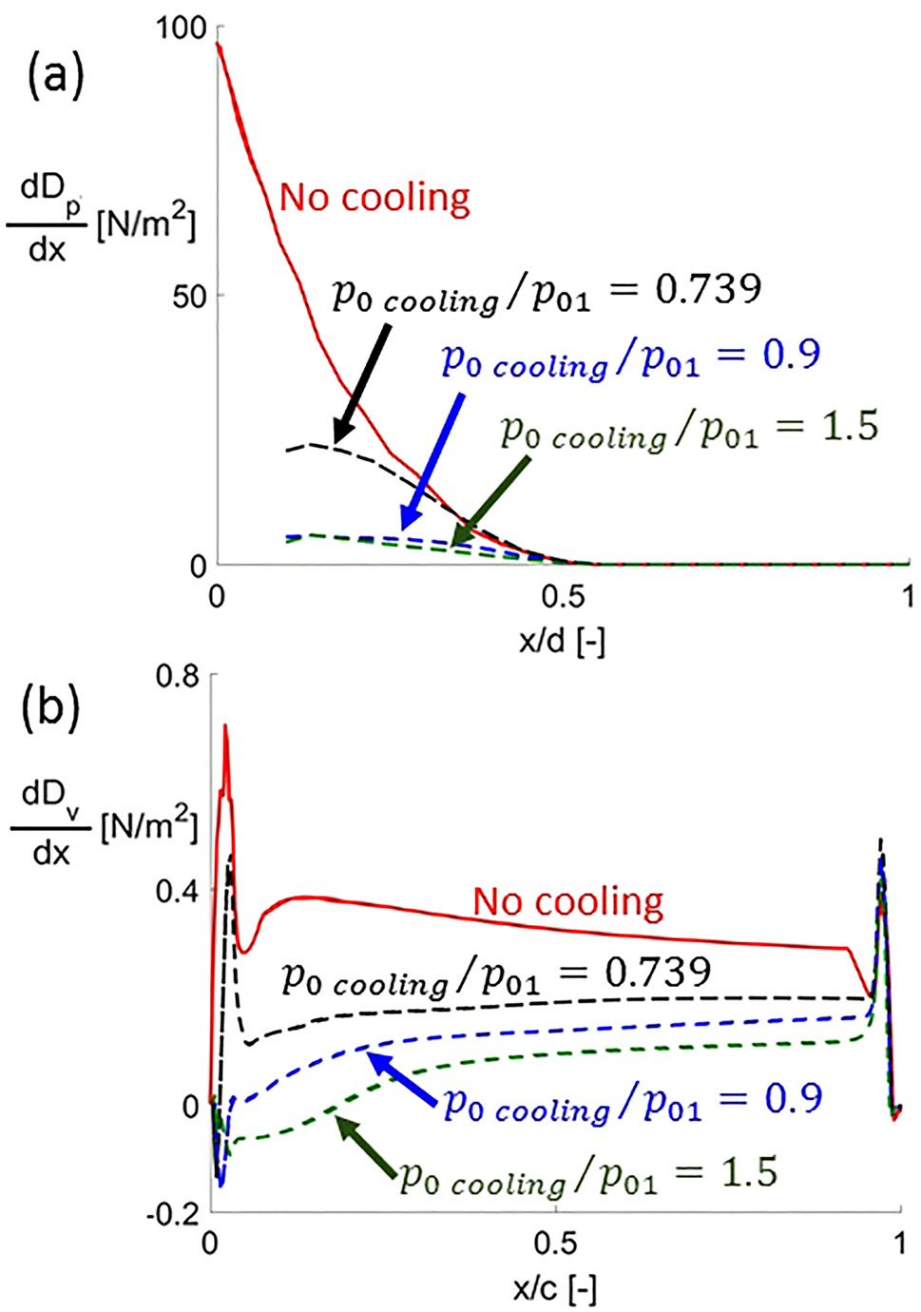
Local (**a**) pressure drag distribution near the leading edge and (**b**) viscous drag distribution along the airfoil wall without actuation and under different steady blowing conditions.

The effects of leading edge injection on the local distributions of pressure and viscous drag are shown in Fig. 9a,b, respectively, for the same conditions depicted in Fig. 8. These local pressure and viscous drag distributions are obtained by projecting on the axial direction the force locally created by pressure and wall shear stress on the airfoil. The pressure drag distributions are focused on the leading edge vicinity, non-dimensionalizing the axial distance with the leading edge diameter. For the uncooled case, the pressure drag distribution has its maximum at the leading edge due to the maximum value of the pressure along the airfoil’s wall at the stagnation point. It decays from there, reaching a 0 value at the transition from the leading edge to the straight part of the airfoil ($x/d = 0.5$). When the cold flow is ejected at the leading edge, the stagnation point of the flow is not at the leading edge surface but leads to a saddle point further upstream, where the main flow and the coolant jet collides (see point E in Fig. 7). As $p_{0\,cooling}$ is increased, the pressure drag values further decrease since the increase in the recirculated area leads to lower pressure, hence lower pressure drag. However, once the supersonic injection is reached, the differences in local pressure drag are minor, leading to a lower contrast between the distributions corresponding to $p_{0\,cooling} /p_{01} = 0.9$ and 1.5 as observed in Fig. 9a.

The viscous drag distributions depicted in Fig. 9b mimic the behaviors observed in the wall shear stress signatures shown in Fig. 8. For the case without actuation, it starts from 0 at the leading edge going immediately through a peak. After that, it has a local minimum before transitioning to a smooth local maximum and decay. At the trailing edge region, it presents a sudden drop from the previous gradual decay and a peak before going back to 0. This fluctuating behavior occurring at the trailing edge is due to the unsteady flow detachment at the base region occurring in a periodic alternating pattern between the lower and the upper side of the airfoil. The distortion travels upstream within the subsonic part of the boundary layer, which justifies the fluctuations in the wall shear stress. As coolant is injected at the leading edge, viscous drag is reduced along the complete airfoil. Moreover, at the leading edge area, the negative shear stress created by the recirculation regions under supersonic injection conditions leads to negative viscous drag values, therefore, thrust is created.

**Figure 10:**
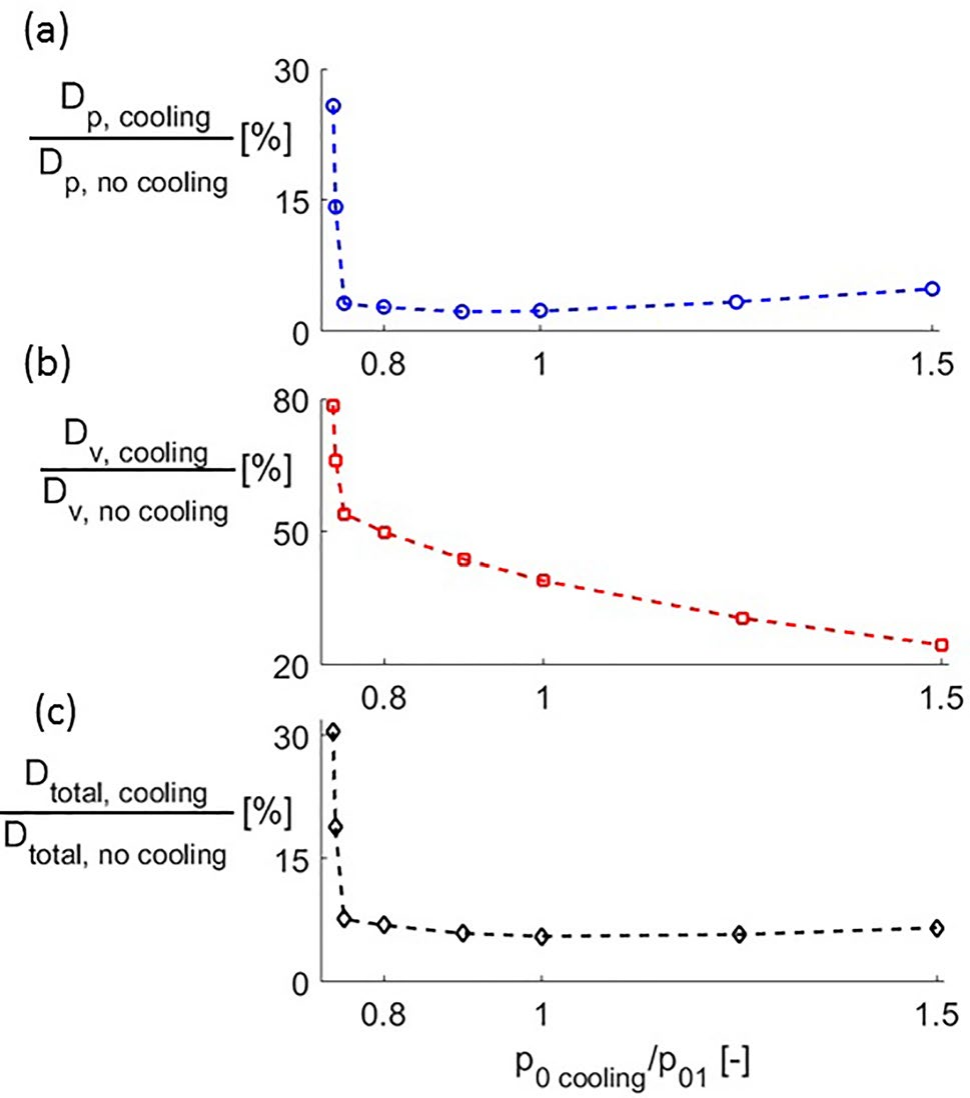
(**a**) Pressure, (**b**) viscous and (**c**) total drag reduction under cooling injection conditions.

Figure 10a–c present the overall pressure, viscous and total drag calculated by integrating along the airfoil. In these figures, the drag is non-dimensionalized by the drag with no cooling. For subsonic injection, as cooling total pressure increases both pressure and viscous drag decrease, causing total drag to decrease as well. In the supersonic injection regime, pressure drag firstly remains unchanged and increases slightly as $p_{0\,cooling}$ is increase beyond $p_{0\,cooling} /p_{01} = 1$.

The maximum reduction in pressure and viscous drag are 97 and 75%, found for $p_{0\,cooling} /p_{01} = 0.9$ and 1.5, respectively. Due to the higher absolute value of the pressure component, the maximum total drag reduction is 94%, for $p_{0\,cooling} /p_{01} = 1.0$.

**Figure 11:**
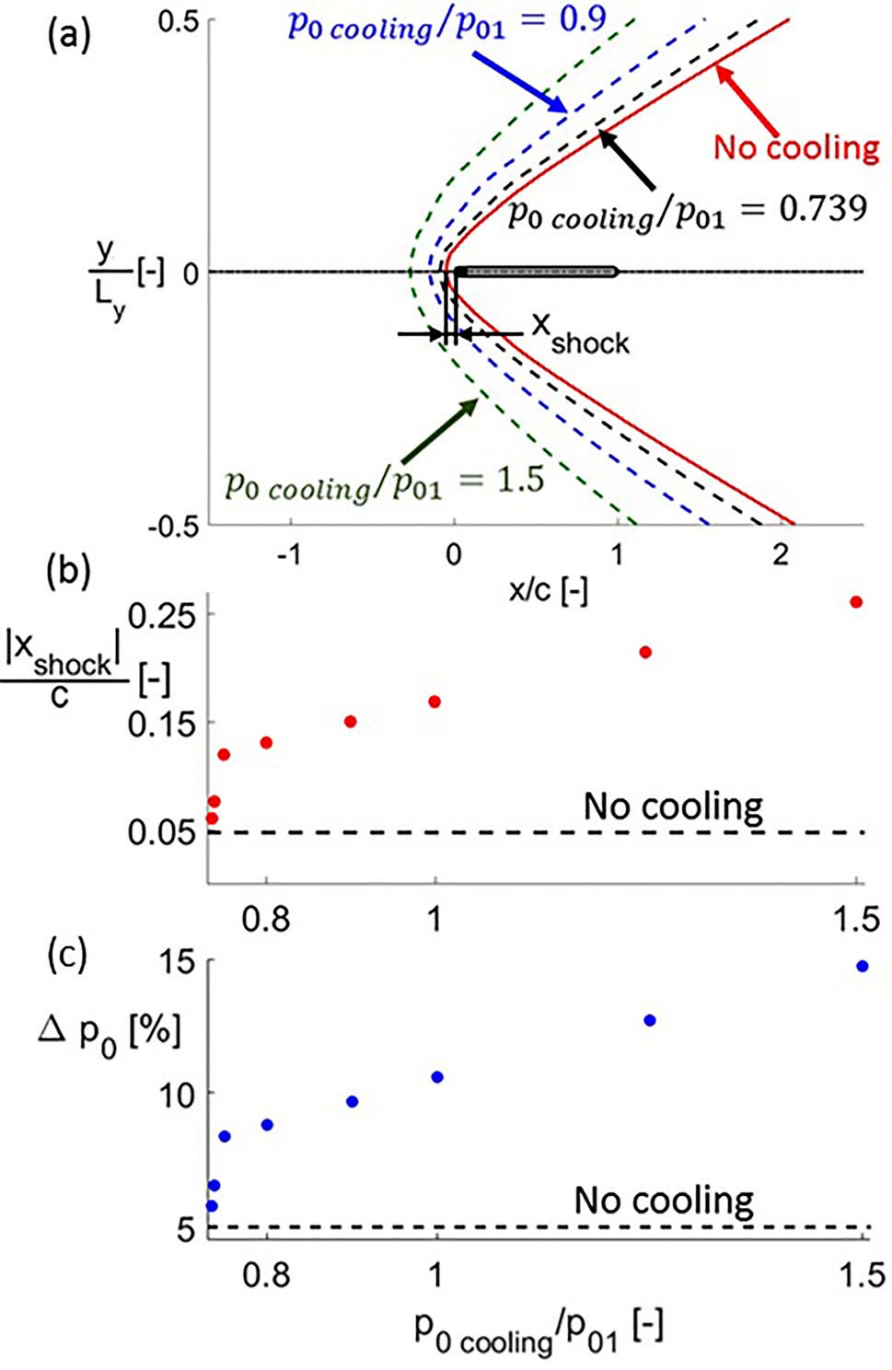
Bow shock shape (**a**), distance from the leading edge (**b**) and total pressure loss (**c**) for different cooling total pressures.

The effect of the injection on the leading edge shock shape can be observed in Fig. 11, graph (a) shows the geometry of the complete shock for different cooling total pressures. Figure 11b represents the distance between the shock and the leading edge along the stagnation line (non-dimensionalized by the airfoil chord) for different cooling total pressures (normalized with the inlet total pressure). As coolant is injected, the momentum of this colder flow jet pushes the bow shock upstream, away from the leading edge. The higher the total pressure of this coolant jet, the higher its momentum, and therefore the further upstream it moves the bow shock. Additionally, the higher momentum rate of the coolant jet with increasing $p_{0\,cooling}$ leads to higher angles of the bow shock relative to the horizontal; thus, the bow shock becomes less oblique. Therefore, increasing the coolant total pressure, causes a higher intensity of the shock, increasing the total pressure losses across the domain. Figure 11c displays this total pressure loss, $\Delta p_{0}$, defined in Eq. (4), where $p_{0}$ represents total pressure and the subindices 1 and 2 are associated to inlet and outlet quantities, respectively.

4 $$ \Delta p_{0} = 1 - \frac{{p_{02} }}{{p_{01} }} $$

The data shown in Fig. 11 corresponds to a dimensionless cooling total temperature of $T_{0\,cooling} /T_{01} = 0.5$. The variation of the cooling total temperature has a negligible effect on the total pressure losses with respect to the one caused by $p_{0\,cooling}$.

The change in the slope in Fig. 11b,c occurs when transitioning to supersonic ($p_{0\,cooling} /p_{01} \ge 0.75$) injection. This slope is steeper for the subsonic injection range, showing a higher sensitivity of $\Delta p_{0}$ with respect to the cooling injection total pressure for these cases.

### Effect on thermal behavior

**Figure 12:**
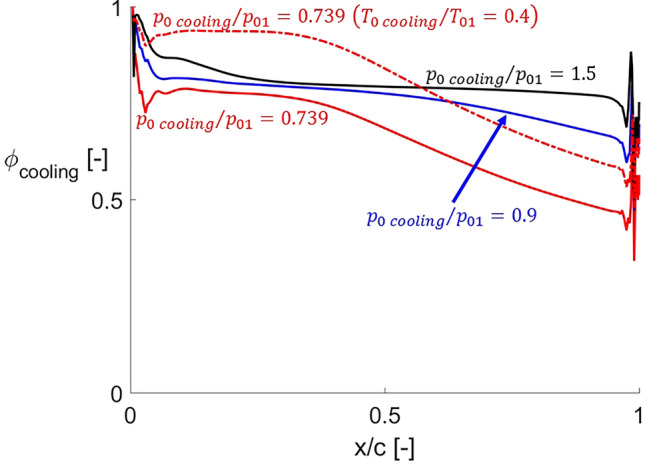
Cooling effectiveness distributions for different cooling total pressures and temperatures.

In order to analyze the effect of the cooling injection on the thermal performance of the airfoil, Fig. 12 shows the isothermal cooling effectiveness along the airfoil as defined in Eq. (5). It represents the ratio between the local heat flux alleviation with cooling and the local heat flux when no cooling is applied^23^.

5 $$ \phi_{cooling} = 1 - \frac{{q_{cooling} }}{{q_{no\,cooling} }} $$

All cooling effectiveness distributions have their maximum at the leading edge, where the cooling is injected, with values close to unity. Immediately downstream, cooling effectiveness suddenly drops towards a plateau. For the lowest cooling total pressure, a second decay is observed. As the cooling total pressure is increased, this decay is less abrupt and occurs further downstream. It is even suppressed for $p_{0\,cooling} /p_{01} = 1.5$. This behavior occurs because higher coolant total pressures imply higher momentum of the coolant jet, which allows the jet to propagate its effect further downstream along the airfoil, better protecting a larger portion of it. The increase in coolant total pressure leads to higher cooling effectiveness values along the complete airfoil. The minimum cooling effectiveness value is found at the trailing edge region, being 0.34 for $p_{0\,cooling} /p_{01} = 0.739$ and 0.47 for $p_{0\,cooling} /p_{01} = 0.9$.

The red solid and dash-dotted lines in Fig. 12 depict the cooling effectiveness distributions at $p_{0\,cooling} /p_{01} = 0.739$ for two different coolant total temperatures. The cooling effectiveness is increased for lower coolant temperatures along the complete airfoil. For this particular distribution, the cooling effectiveness remains in values close to 90% up to the mid-chord. Therefore, the lower cooling temperature helps to better protect the airfoil against the hot incoming flow by lowering down the local driving temperature of the convective heat transfer process.

**Figure 13:**
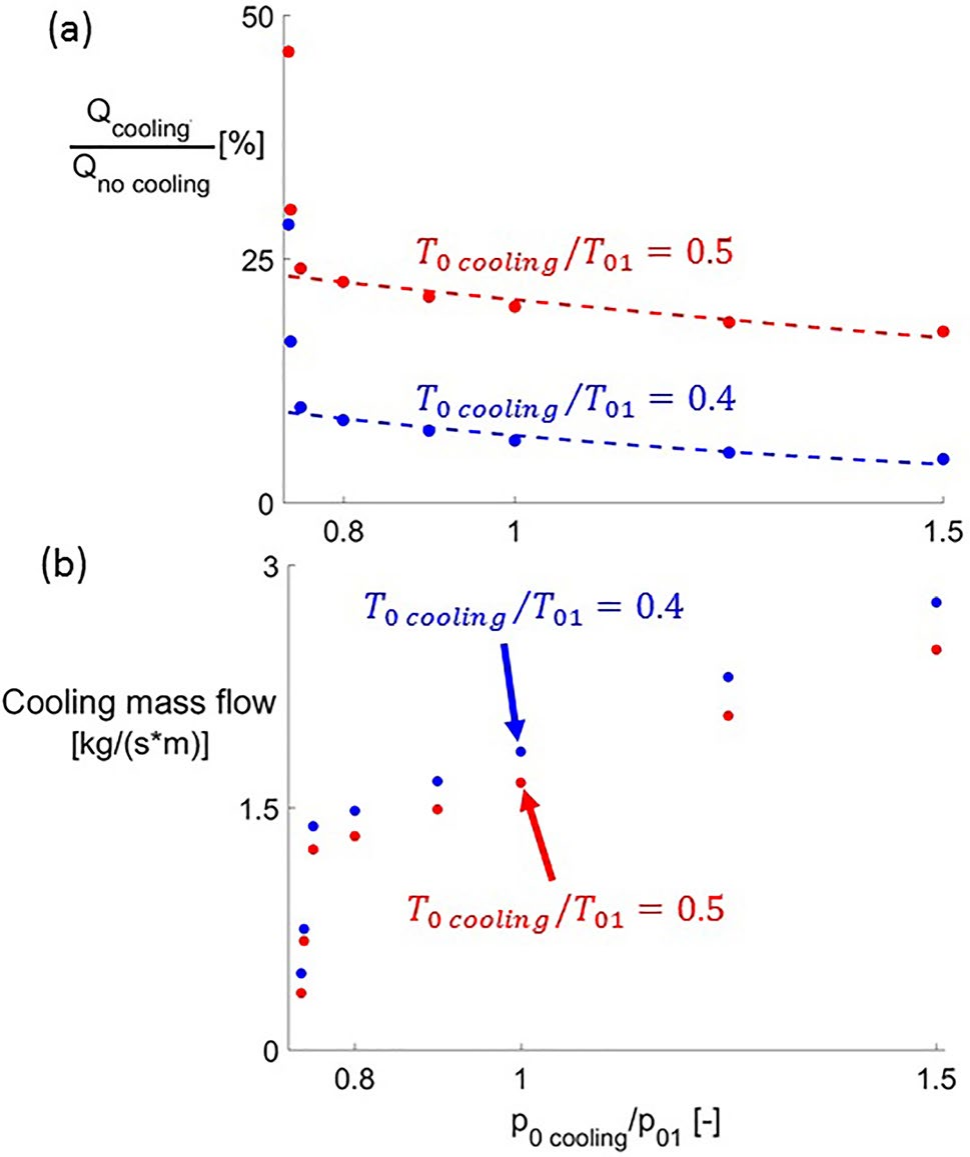
Thermal load alleviation (**a**) and cooling mass flow (**b**) for steady blowing conditions.

The overall thermal load alleviation for different steady cooling conditions is shown in Fig. 13a. This overall thermal load $Q$ is the heat flux integral along the airfoil. In the graph, it is normalized by its value when no cooling is applied. This ratio is represented with respect to $p_{0\,cooling} /p_{01}$ for the two different values of $T_{0\,cooling}$. Decreasing $T_{0\,cooling}$ significantly reduces the thermal load, reaching for $p_{0\,cooling} /p_{01} = 1.5$ and $T_{0\,cooling} /T_{01} = 0.4$ a reduction in $Q$ of 93% with respect to the uncooled configuration. This is the maximum thermal load reduction encountered when injecting in steady conditions in the present study, appearing for supersonic injection. This kind of injection reports larger thermal load alleviations than subsonic one. However, it is also more expensive in terms of the mass flow it requires. e.g. for $p_{0\,cooling} /p_{01} = 0.75$ the cooling mass flow is $50 \%$ less than the mass flow required for $p_{0\,cooling} /p_{01} = 1.5$. The cooling mass flows injected under different cooling boundary conditions are depicted in Fig. 13b. The thermal performance of the airfoil, as well as the aerodynamic one, is more sensitive to $p_{0\,cooling}$ variations for subsonic injection conditions than for supersonic ones.

The expressions describing the exponential laws for the heat alleviation for supersonic injection conditions are given in Eqs. @@@(6a, 6b) for $T_{0\,cooling} /T_{01} = 0.5$ and $0.4$, respectively. Having these two laws allows the extrapolation of the thermal load alleviation that would occur at other cooling boundary conditions, facilitating the work of future designers without the need of running new simulations.

6a $$ \frac{{Q_{cooling} }}{{Q_{no\,cooling} }} = 0.32 e^{{ - 0.42 \left( {p_{0\,cooling} /p_{01} } \right)}} $$

6b $$ \frac{{Q_{cooling} }}{{Q_{no cooling} }} = 0.21 e^{{ - 1.12 \left( {p_{0 cooling} /p_{01} } \right)}} $$

### Cooling injection port size effect

**Figure 14:**
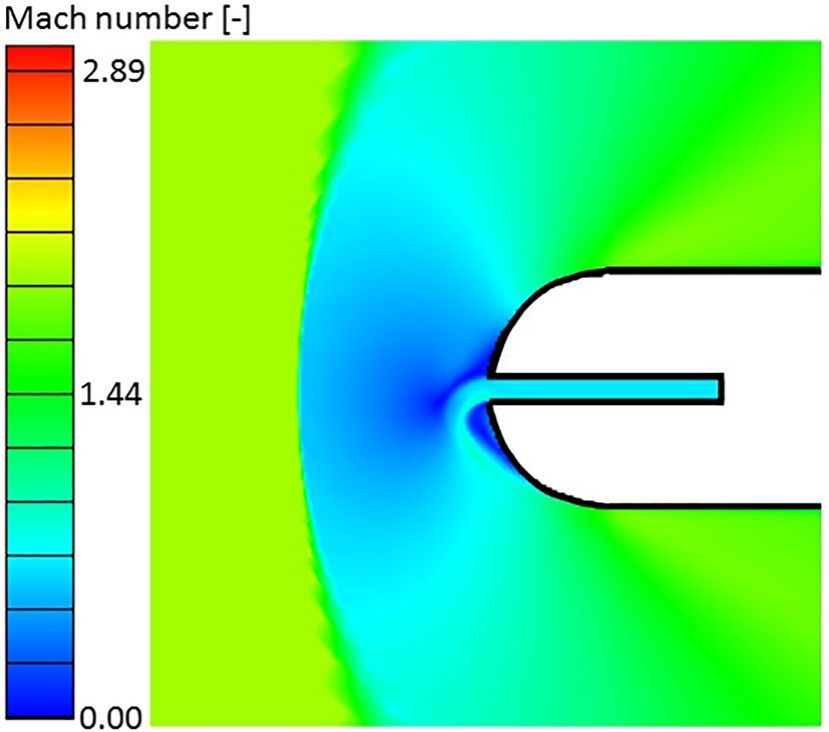
Leading edge region Mach contour with Coanda effect flow topology due to cooling injection.

Three different cooling rates were studied with a cooling injection port diameter $d_{cooling} = d/10$. The case at $p_{0\,cooling} /p_{01} = 0.8$ corresponds to a subsonic injection while the cases at $p_{0\,cooling} /p_{01} = 1.5$ and $2$ yield supersonic injections. Interestingly, the two cases with lower $p_{0\,cooling}$ feature a Coanda effect, which makes the flow topology at the leading edge non-symmetric. Therefore, there is a threshold in $p_{0\,cooling} /p_{01}$ below which this Coanda effect appears. The leading edge region Mach number contour for the case at $p_{0\,cooling} /p_{01} = 0.8$ and $T_{0\,cooling} /T_{01} = 0.5$ is given in Fig. 14. As seen in this image, despite the complete symmetry in the problem geometry and boundary conditions, the cooling flow deviates to one side, creating a non-symmetric configuration. For the two cases in which the Coanda effect has been found the jet deviates to its left as it departs the injection cavity. This is the first time in the literature that such a bifurcation phenomenon has been identified in a leading edge injection configuration as the one studied herein.

Coanda effect was observed before in symmetric injection configurations, although not for leading edge injection in a supersonic free stream as it was identified in this study. e.g. at the trailing edge Martinez-Cava et al.^15^ identified it for injection at the trailing edge. At a critical Reynolds number, a bifurcation exists which causes the flow topology to transition from a symmetric to an asymmetric configuration. In this asymmetric topology, one of the separation regions on the sides of the expansion becomes larger than the other, deflecting the flow towards the small recirculation side. In the present case, the sudden expansion occurring at the injection port exit triggers the Coanda effect under certain injection conditions. For trailing edge injection, Coanda effect may be exploited as a flow control tool^24^. Similarly, Coanda effect appearing at the leading edge may enable flow control tools at the leading edge.

**Figure 15:**
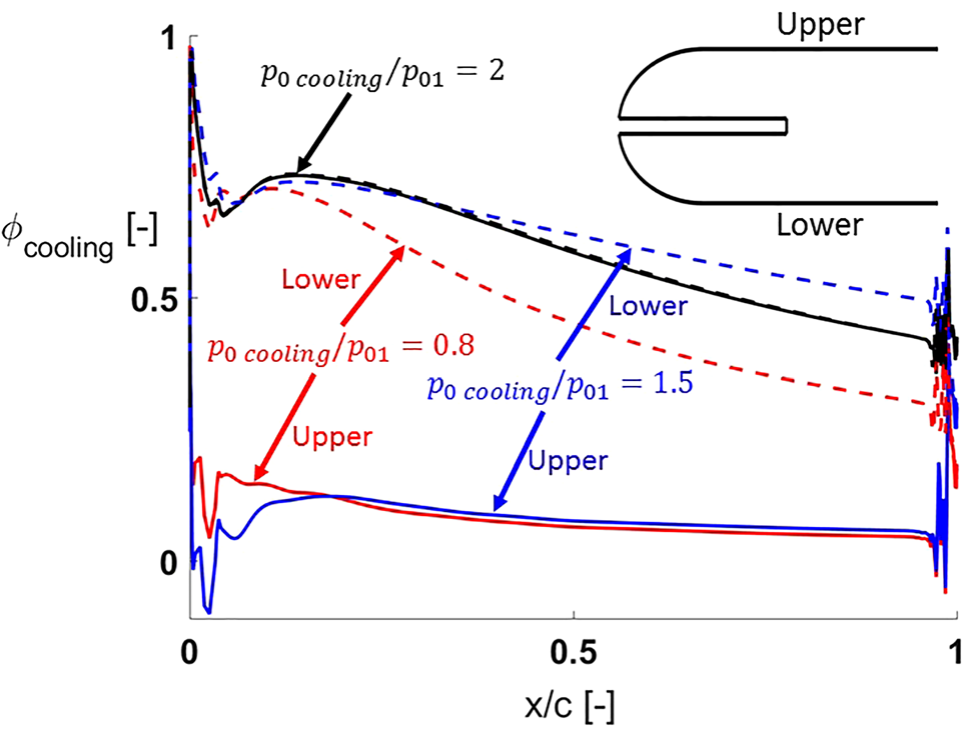
Cooling effectiveness distributions for cases with a cooling injection port size $d_{cooling} = d/10$.

Figure 15 represents the cooling effectiveness distributions along the airfoil for the three different cooling total pressures studied with a cooling injection port diameter $d_{cooling} = d/10$. For each of the three different $p_{0\,cooling} /p_{01}$ values, two different curves are plotted. The solid line corresponds to the upper half of the airfoil while the dashed line corresponds to the bottom one. For $p_{0\,cooling} /p_{01} = 0.8$ and $1.5$, the cooling effectiveness distributions are different, with lower values on the top surface of the airfoil. Hence, that side is less protected by the cooling injected at the leading edge. As cooling total pressure is increased to $p_{0\,cooling} /p_{01} = 2$, the Coanda effect disappears and both curves collapse to a single one.

Cooling effectiveness for $p_{0\,cooling} /p_{01} = 0.8$ and $1.5$ on the side towards which cooling is not deflected is close to 0 along most of the airfoil. For $p_{0\,cooling} /p_{01} = 0.8$, it even has a small region where it becomes negative close to the leading edge. This means that heat flux in the particular region is higher for the cooled than for the uncooled configuration.

Table 5 summarizes the cooling and aerodynamic performance of the steady leading edge injection for the cases in which the injection port diameter is $d_{cooling} = d/10$. Lower thermal load and drag reductions, and lower total pressure loss are reached with respect to the cases with a port size $d_{cooling} = d/2$. Regarding the thermal load alleviation, the two lower cooling total pressures give similar values, when the coolant total pressure achieves $p_{0\,cooling} /p_{01} = 2$, the symmetric flow topology gives thermal protection to both sides of the airfoil and the thermal load is significantly reduced with respect to lower cooling total pressures.

**Table 5:** Cooling performance summary of $d_{cooling} = d/10$ diameter injection port cases.

$\frac{{p_{0\,cooling} }}{{p_{01} }}\,( - )$	$\frac{{Q_{Cooling} }}{{Q_{No\,cooling} }}\,(\% )$	$\frac{{D_{total\,cooling} }}{{D_{total\,no\,cooling} }}\,(\% )$	$\Delta p_{0} \,(\% )$
0.8	68.13	75.78	5.46
1.5	62.35	49.43	5.82
2	37.13	29.12	5.92

### Coanda effect parametric study

To further understand the nature of the Coanda effect appearing for certain injection conditions, a more in-depth parametric study was conducted. This study involved the cooling Reynolds and Mach numbers, the impact of flow angle incidence. Nine cases were considered to evaluate the cooling Reynolds (${\text{Re}}_{dc}$) and Mach number ($M_{c}$) effects. The values of these parameters and the total pressure and temperatures imposed at the cooling injection are listed in Table 6. The ${\text{Re}}_{dc}$ was varied between $0.5 \times 10^{4}$ and $1.5 \times 10^{4}$ and $M_{c}$ between $0.70$ and $1.00$. Both Reynolds and Mach numbers were evaluated at the cooling injection pressure inlet boundary. The Reynolds number is based on the cooling injection slot width ($d_{cooling}$).

**Table 6:** Cooling Reynolds and Mach numbers and the corresponding coolant total pressures and temperatures.

${\text{Re}}_{dc} \,( - )$	$M_{c} \,(\% )$	$\frac{{p_{0\,cooling} }}{{p_{01} }}\,( - )$	$\frac{{T_{0\,cooling} }}{{T_{01} }}\,(\% )$
$1.00 \times 10^{4}$	0.85	0.915	0.334
$1.00 \times 10^{4}$	0.70	0.802	0.272
$1.00 \times 10^{4}$	1.00	2.022	0.720
$0.50 \times 10^{4}$	0.85	0.940	0.645
$1.50 \times 10^{4}$	0.85	0.898	0.231
$0.50 \times 10^{4}$	0.70	0.813	0.519
$1.50 \times 10^{4}$	0.70	0.800	0.192
$0.50 \times 10^{4}$	1.00	2.560	1.750
$1.50 \times 10^{4}$	1.00	2.175	0.500

To evaluate the symmetry or presence of the Coanda effect on the leading edge flow topology, we use the ratio of the mean pressures in the two sides of the front part of the leading edge. As the numerator, we use the highest value, which could be the lower or upper side of the airfoil, divided by the smallest mean static pressure level. Hence, the mathematical expression for this ratio is $max\left\{ {p_{U} ,{ }p_{L} } \right\}/min\left\{ {p_{U} ,{ }p_{L} } \right\}$. The mean static pressures $p_{U}$ and $p_{L}$ were evaluated between the centerline of the leading edge geometry and the position corresponding to $45^\circ$ on the circular arcs defining the leading edge on each half of the airfoil.

**Figure 16:**
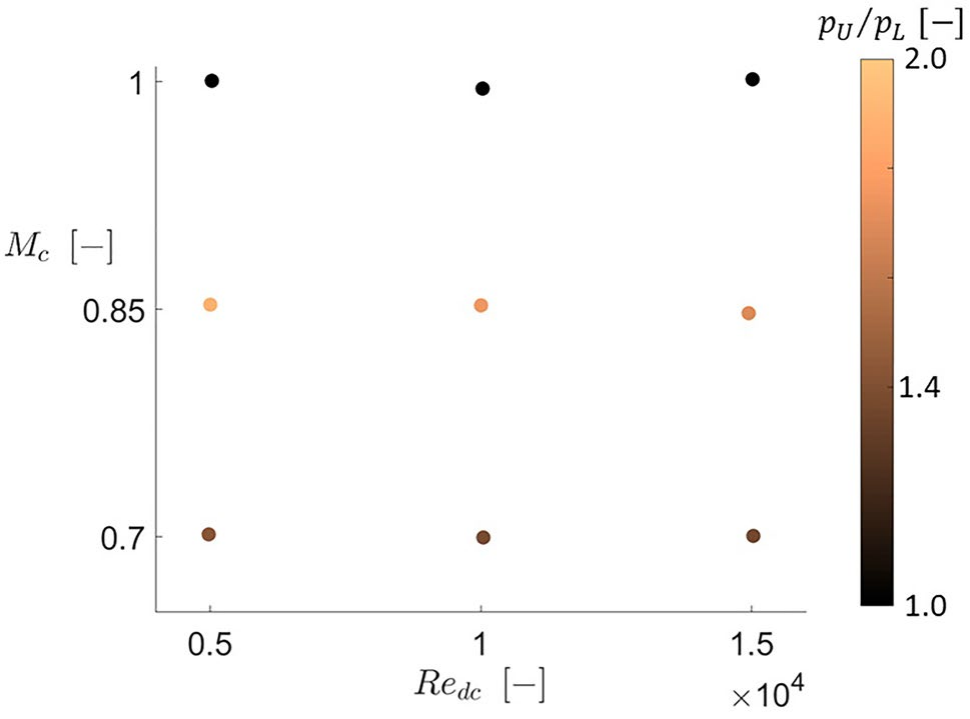
Coanda parametric study cooling Reynolds and Mach number map colored with the ratio of mean static pressure on both sides of the leading edge.

Figure 16 represents the points evaluated when varying the cooling Reynolds and Mach numbers. The first parameter is plotted on the horizontal axis and the second parameter on the vertical one. The points represented in this graphic are colored by the $p_{U} /p_{L}$ ratio. No significant Reynolds effects were observed for the range studied. However, symmetric configurations appeared for the highest cooling Mach number studied ($M_{c} = 1.00$) while the Coanda effect causing an asymmetric flow topology appeared for lower values of this parameter. Hence, compressibility plays an essential role in the leading edge flow topology.

**Figure 17:**
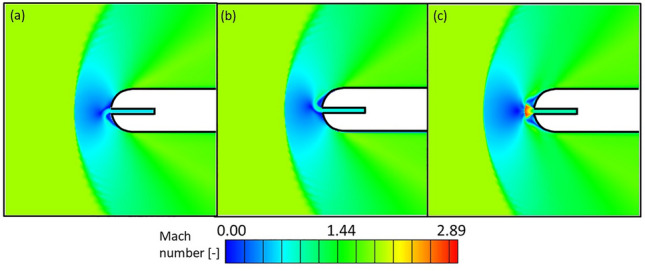
Leading edge region Mach number contours for ${\text{Re}}_{dc} = 1.00 \times 10^{4}$ and $M_{c} = 0.70$ (**a**), 0.85 (**b**) and 1.00 (**c**).

The Mach number contours on the leading edge vicinity for different cooling injection Mach numbers are depicted in Fig. 17. Graphics (a) and (b) show the asymmetric flow topologies appearing for low Mach numbers. In contrast, contour (c) shows the symmetric configuration when sonic conditions are reached at the cooling injection.

**Figure 18:**
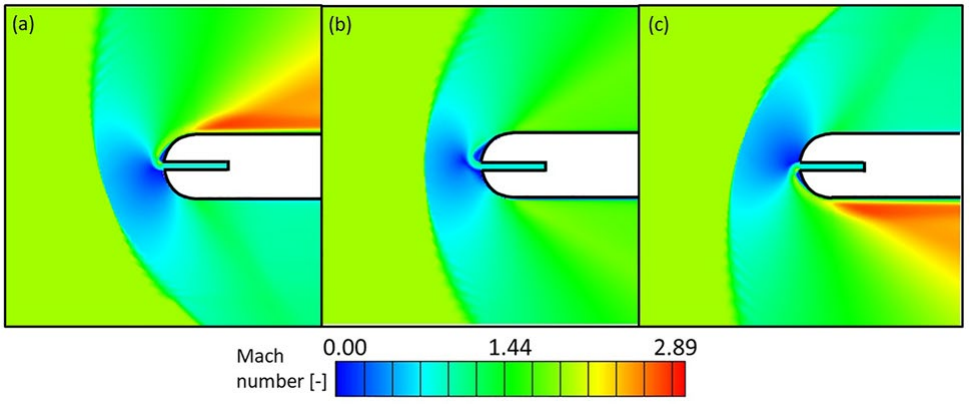
Leading edge region Mach number contours for ${\text{Re}}_{dc} = 1.00 \times 10^{4}$ and $M_{c} = 0.85$, $\alpha_{1} = - 20$ (**a**), 0 (**b**) and 20° (**c**).

The effect of the mainstream flow angle, $\alpha$, on the leading edge flow topology was evaluated two different cases: the one with ${\text{Re}}_{{{\text{dc}}}} = 1.00 \times 10^{4}$ and ${\text{M}}_{{\text{c}}} = 0.85$ and the one with ${\text{Re}}_{{{\text{dc}}}} = 1.00 \times 10^{4}$ and ${\text{M}}_{{\text{c}}} = 1.00$. These cases correspond to a Coanda and symmetric flow topology when the mainstream is completely axial, respectively. In both cases, the inlet flow angle ($\alpha_{1}$) was varied between $- { }20$ and $+ { }20^\circ$, where positive flow angles mean that the flow arrives at the leading edge from the top part of the 2D domain given in Fig. 2. Figure 18 shows the Mach number contours for $\alpha_{1} = { } - { }20$, $0$ and $20^\circ$. In the three cases, ${\text{Re}}_{dc} = 1.00 \times 10^{4}$ and $M_{c} = 0.85$. Graphic (b) depicts the Coanda effect appearing for the axial flow conditions under these cooling Reynolds and Mach numbers. The variation of the flow angle from axial conditions modifies the bow shock shape and the cooling direction. The bow shock rotates to face the incoming free stream while the cooling flow is diverted to the opposite side to the incoming flow. Once this coolant flow rotates around the curved leading edge and arrives at the flat part of the airfoil, it accelerates through an expansion fan, reaching Mach numbers of up to $3$.

The possible appearance of spanwise effects was evaluated running a 3D RANS case in which the previously considered 2D domain was extruded one chord length, $60\;{\text{mm}}$. The cooling boundary conditions considered for this case are those corresponding to ${\text{Re}}_{dc} = 1.00 \times 10^{4}$ and $M_{c} = 0.85$. The limits of the extrusion were considered slip adiabatic walls. The Coanda effect occurs for this case, making the leading edge flow topology non-symmetric. Furthermore, the topology is identical at different positions in the spanwise direction.

## Unsteady blowing effect

**Figure 19:**
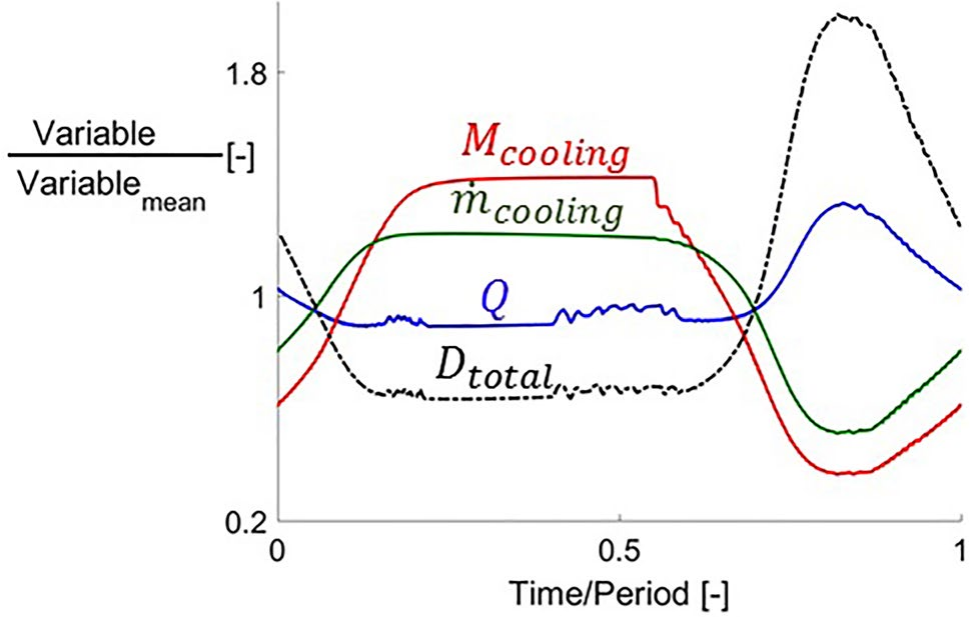
Thermal load and total drag evolutions with respect to time together with cooling mass flow and injection Mach number.

The evolution of the aerodynamic and thermal parameters together with the flow field reported so far for steady blowing conditions were analyzed for fluctuating cooling conditions. Figure 19 represents the evolution of cooling injection Mach number, mass flow, total drag and thermal load as a function of non-dimensional time from $0$ to $1$ period. The data shown in Fig. 19 corresponds to $T_{0\,cooling} /T_{01} = 0.5$ and a fluctuation frequency of $10\,{\text{Hz}}$ in $p_{0\,cooling}$.

Cooling Mach number and mass flow increase as $p_{0\,cooling}$ does, from $t/P = 0$ to $0.15$. Then the injection port gets choked, reaching $M_{cooling} = 1$ and the different variables remain constant until the coolant total pressure decreases again. During this portion of the period with high $p_{0\,cooling}$ the thermal load over the airfoil decreases, reaching its minimum. Two intervals in which $Q$ and drag present high frequency fluctuations occur immediately before the cooling port gets choked and unchoked. In between them, both variables remain constant while the injection cavity is choked. During the second half of the period, when the cooling total pressure is decreased below its mean value, $M_{cooling}$ and $\dot{m}_{cooling}$ decrease as well. The thermal load has the opposite behavior during this part of the period, reaching its maximum value. Even though the peak-to-peak amplitude of the fluctuations in $p_{0\,cooling}$ is only $1.9 \%$ of its mean value, peak-to-peak amplitudes of $106 \%$ for $M_{cooling}$, $71 \%$ for $\dot{m}_{cooling}$ and $44 \%$ for $Q$ and $137 \%$ for total drag were obtained.

**Figure 20:**
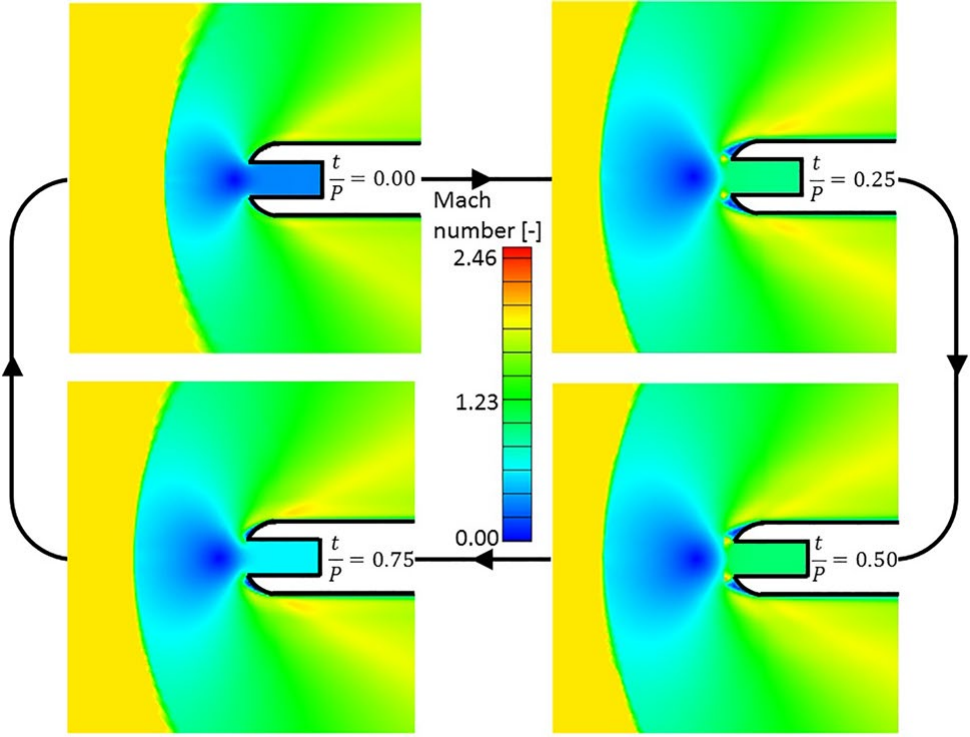
Time snapshots of the Mach number contour at the leading edge region throughout a fluctuation period for $T_{0\,cooling} /T_{01} = 0.5$, $f = 10\,{\text{Hz}}$.

Figure 20 depicts four different time snapshots of the Mach number contour at the leading edge vicinity for $T_{0\,cooling} /T_{01} = 0.5$ and $f = 10\,{\text{Hz}}$. At the beginning of the period, $t/P = 0$, a subsonic injection flow field can be observed, with low subsonic Mach number at the cooling injection cavity. As total pressure is increased, supersonic injection already appears for $t/P = 0.25$ with two supersonic pockets at the exit of the cooling cavity and two separation regions on the sides of the injection. The larger momentum of the jet for this supersonic injection pushes the bow shock further away from the leading edge. Supersonic injection can still be observed for $t/P = 0.50$. As the cooling total pressure is decreased throughout the second half of the fluctuation period, subsonic injection is retrieved again for $t/P = 0.75$, in this case with a higher Mach number at the injection than the one at the beginning of the period.

**Figure 21:**
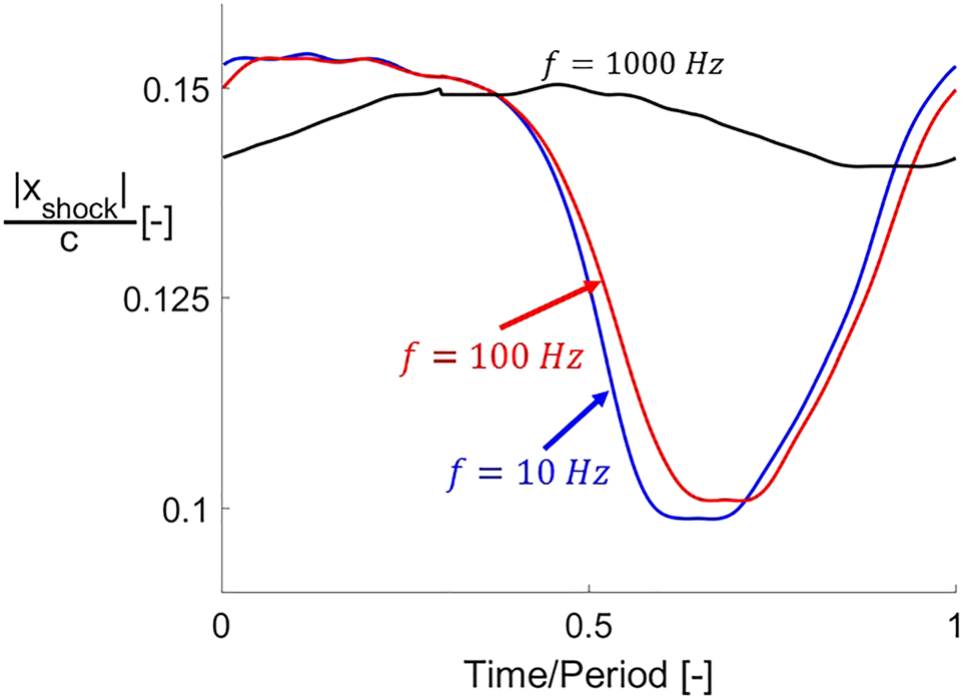
Bow shock distance from leading edge evolutions with respect to time for three different cooling total pressure fluctuation frequencies.

The effects of the cooling total pressure fluctuation on the bow shock position are shown in Fig. 21. For the two lower frequencies ($10$ and $100\,{\text{Hz}}$), the position of the shock has its maximum during the first half of the fluctuation since the higher total pressure, and hence momentum, of the leading edge jet pushes it further away. During the second half of the fluctuation, during which subsonic injection occurs, the lower momentum of the jet makes the bow shock approach the leading edge. For the higher frequency of $100\,{\text{Hz}}$, the amplitude of the fluctuation in the shock position throughout the period is reduced.

**Figure 22:**
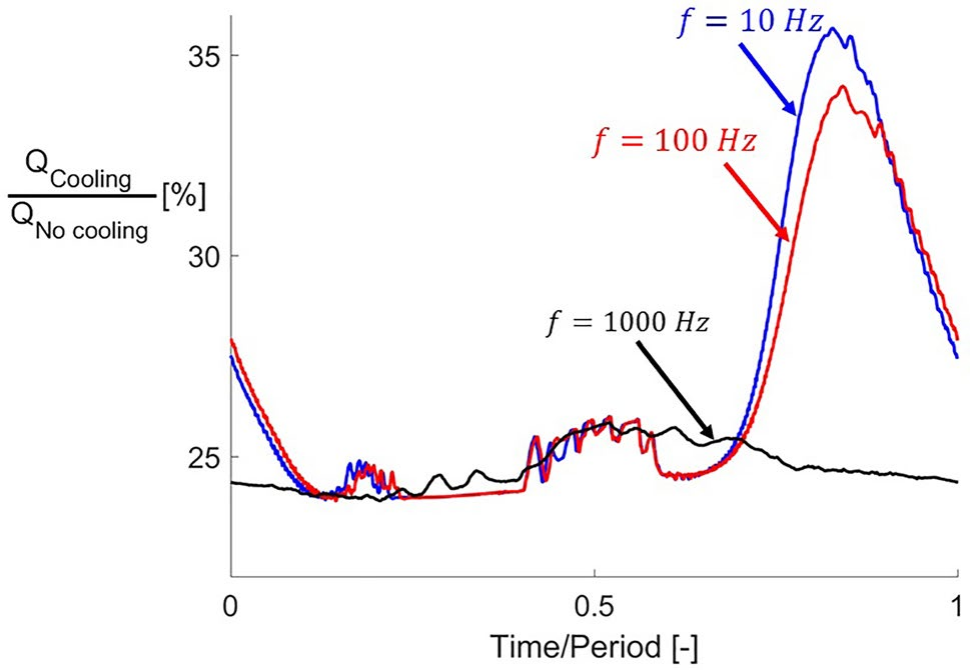
Thermal load alleviation evolutions with respect to time for three different cooling total pressure fluctuation frequencies.

A similar behavior can be observed in the unsteady evolution of the thermal load alleviation when fluctuating the cooling total pressure at different frequencies. The unsteady evolution of this variable is depicted in Fig. 22 for the three different frequencies analyzed in this study. The data presented in this graph corresponds to $T_{0\,cooling} /T_{01} = 0.5$. For fluctuation frequencies of $10$ and $100\,{\text{Hz}}$ (blue and red curves, respectively), the thermal load alleviation displays the same behavior shown in Fig. 19). When the fluctuation frequency is increased, the system becomes more insensitive to those fluctuations. At $1000\,{\text{Hz }}$ the thermal load temporal evolution is almost flat compared with the two lower frequency cases. The system reaches a quasi-steady state when the cooling fluctuation reaches this high frequency, becoming insensitive to the varying inlet cooling. This finding agrees with previously documented phenomena described in supersonic passages^2^ exposed to fluctuating inlet conditions, note that the previous research considered uncooled supersonic airfoils. These authors identified that the passage aerodynamic behavior reaches a quasi-steady state when the inlet conditions vary at high frequency.

Table 7 shows the mean thermal load alleviation and cooling mass flows retrieved from the unsteady simulations in this study. The pulsating may deliver mean thermal load alleviations close to the maximum ones achieved with steady supersonic injection. Nevertheless, the mean cooling mass flow employed with a pulsating strategy is lower than for steady supersonic injections.

**Table 7:** Thermal load alleviation summary for unsteady cooling cases.

$\frac{{T_{0\,cooling} }}{{T_{01} }}\,( - )$	$f\,(Hz)$	$\left( {\frac{{Q_{Cooling} }}{{Q_{No\,cooling} }}} \right)_{mean} \,(\% )$	$\dot{m}_{cooling\,mean}$ $({\text{kg}}/\left( {{\text{s}}*{\text{m}}} \right))$
0.5	10	25.23	1.01
	100	25.70	1.02
	1000	24.68	1.20
0.4	10	10.33	1.13
	100	10.34	1.14
	1000	9.91	1.34

## Conclusions

The effect on the flow topology created by the injection at the leading edge of an airfoil facing a supersonic free stream has been numerically investigated. The diverse shock waves and flow topologies caused by steady subsonic and supersonic injections as well as pulsating ones have been examined. Overall, leading edge injection pushes the bow shock upstream of the leading edge and makes it more normal in its core region for the studied regimes. The induced flow topology modifications lead to reductions in the thermal load over the airfoil as well as its drag, creating however an increase in total pressure loss due to the bow shock intensity increase.

For steady injection, a coolant total pressure threshold has been identified, which separates two distinct injection regimes. For pressures below this threshold the coolant is injected with subsonic conditions while above it, it is injected in supersonic conditions, with the latter providing a higher aerodynamic and thermal effect. Pulsating cooling was evaluated at $10$, $100$ and $1000\,{\text{Hz}}$, revealing a quasi-steady performance at high frequency. Comparing steady and unsteady injections, the steady supersonic creates the largest thermal and aerodynamic modifications, however, the steady subsonic and pulsating ones have also proven significant modifications requiring lower cooling mass flow rates.

The investigation of different injection port sizes, $d_{cooling} = d/2$ and $d/10$, yielded the discovery of a Coanda effect for the smallest one, deflecting the cooling to one side despite the complete symmetry of the problem setup. This feature was associated with a bifurcation phenomenon in sudden expansions in previous literature. However, this is the first time it is documented and analyzed for the injection at the leading edge of a supersonic body. This finding should enable a novel flow control strategy for future supersonic airfoils.

## List of symbols

*A*: Amplitude (–)
Α: Flow angle (deg)
*M*: Mach number (–)
*C*: Chord (m)
*d*: Leading edge diameter (m)
*D*: Drag force per unit length (N/m)
*f*: Frequency (Hz)
*M*: Mach number (–)
$\dot{m}$: Mass flow (kg/(s m))
*p*: Pressure (Pa)
*P*: Period (s)
$\Delta p_{0}$: Total pressure loss (–)
*q*: Heat flux (W/m^2^)
*Q*: Thermal load (W/m)
*t*: Time (s)
*T*: Temperature (K)
*ϕ*: Cooling effectiveness (–)

## Subscripts

0: Total conditions
1: Inlet conditions
2: Outlet conditions
